# Supplementary figures and images for: The exocytosis regulator complexin controls spontaneous synaptic vesicle release in a CAPS-dependent manner at C. elegans excitatory synapses
Source: PLoS Biol. 2025 Feb 6;23(2):e3003023. doi: 10.1371/journal.pbio.3003023 (PMC11838871; doi:10.1371/journal.pbio.3003023)

A

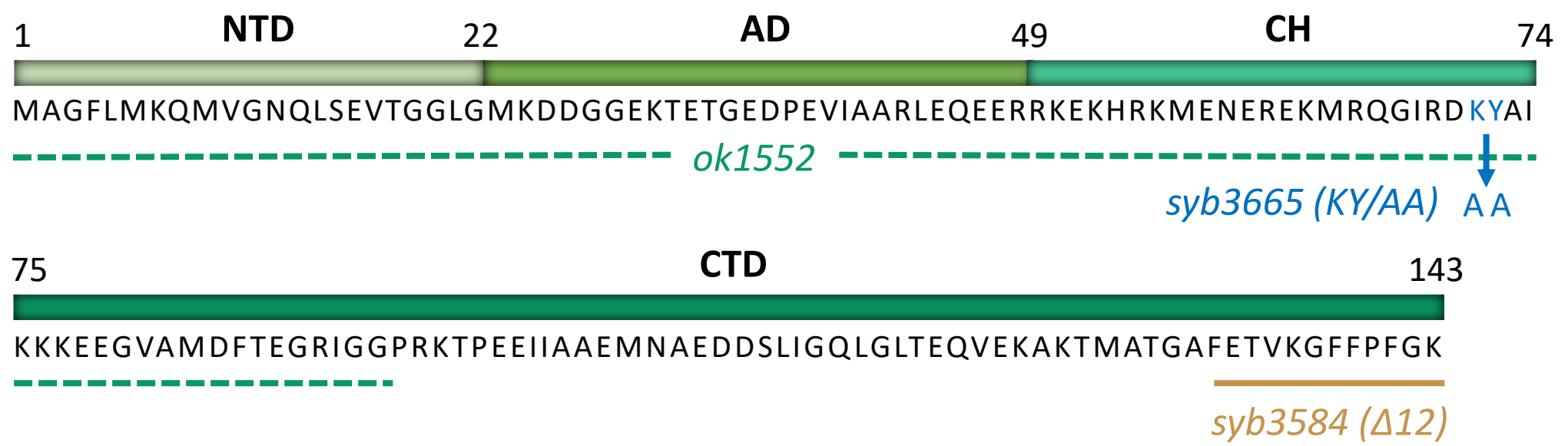

B

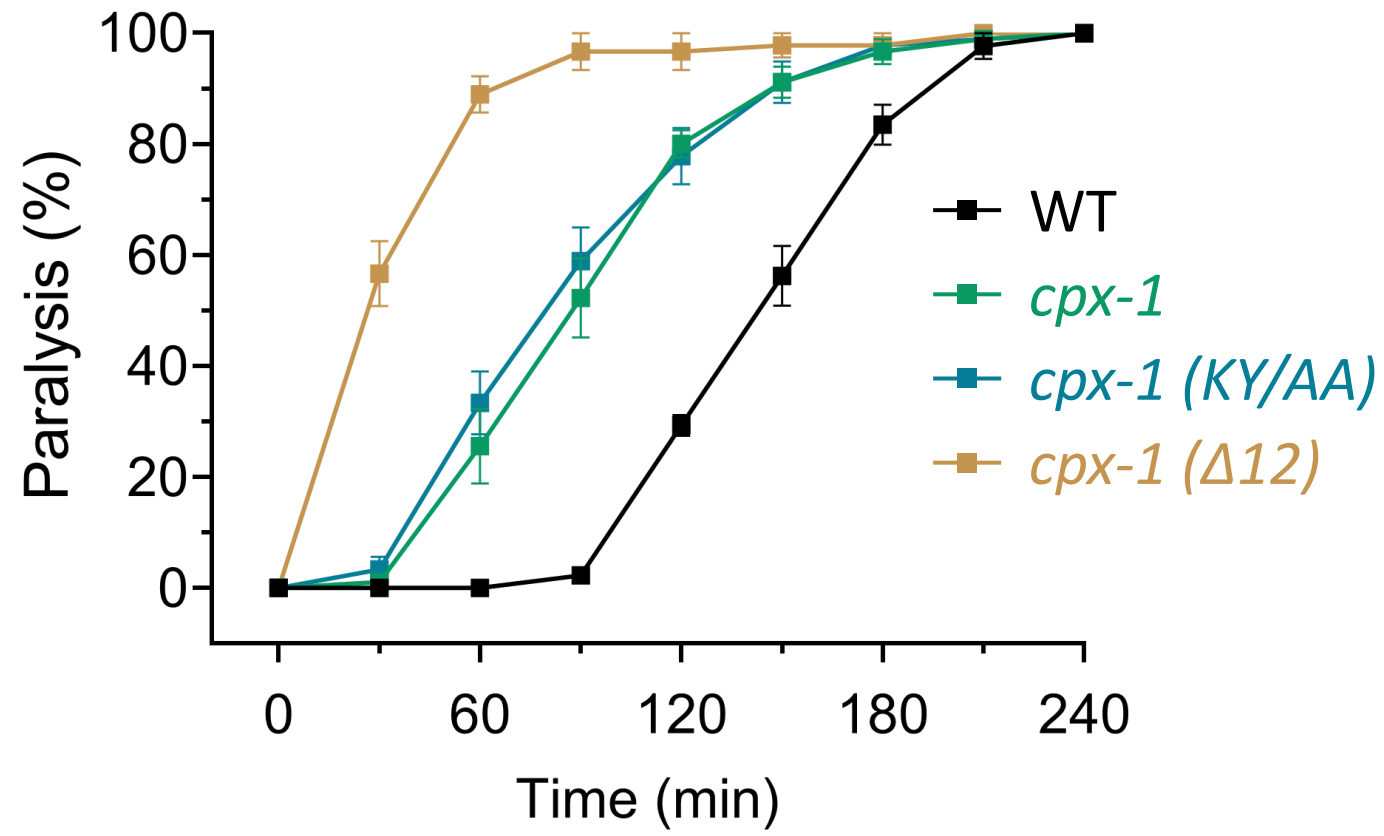

Supplement: S1 Fig — (A) The amino acid sequence of CPX-1, indicating the N-terminal domain (NTD), accessory domain (AD), central α-helix (CH), and C-terminal domain (CTD). Mutation sites of ok1552 (green line), syb3665 (blue stars), and syb3584 (brown line) are labeled. (B) Aldicarb assay for the indicated strains (1 mM aldicarb). Six trials were conducted with 15 worms in each trial. N = 6 independent replicates. All the raw data associated with this figure are available in S1 Data. (PDF) [file pbio.3003023.s001.pdf]

**A**

wild type

*unc-31(e928)*

Rescue

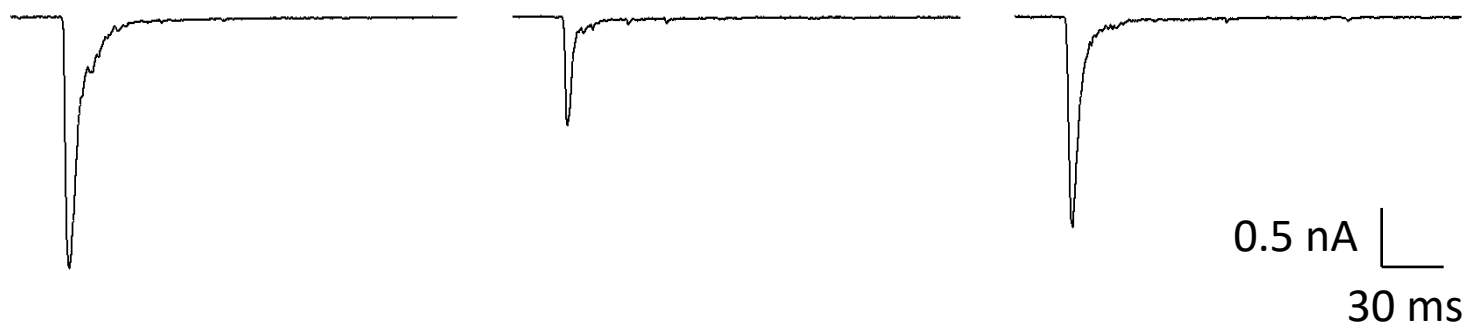**B**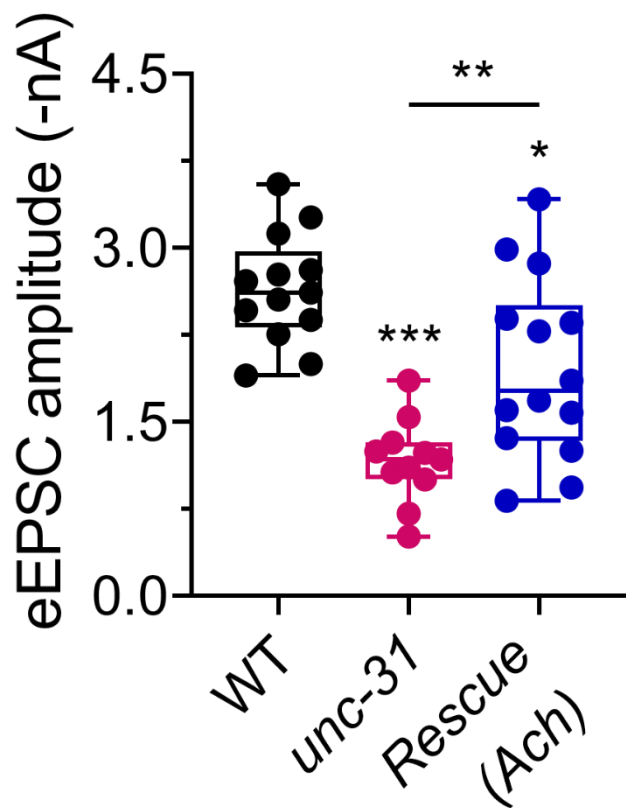**C**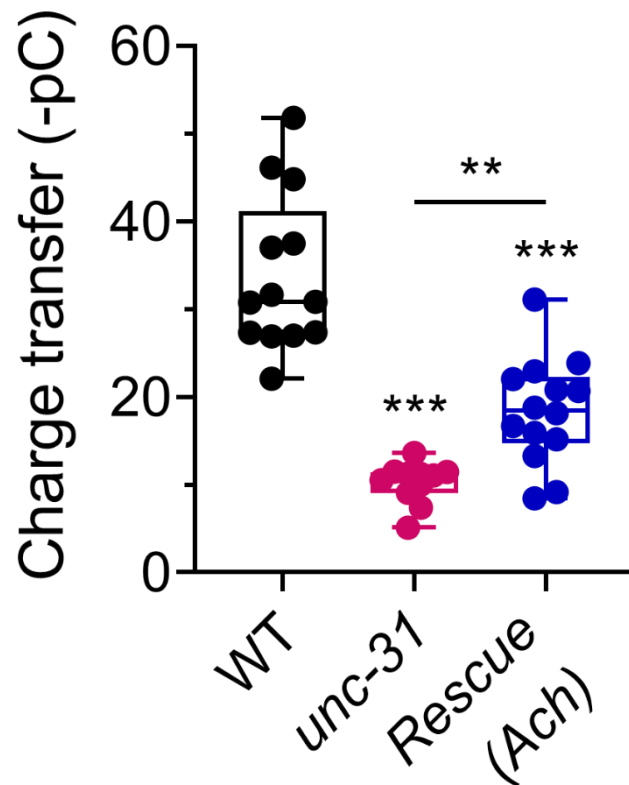

Supplement: S2 Fig — (A) Representative traces of evoked EPSCs recorded from wild-type, unc-31(e928), and rescue strains unc-31(e928); gaaEx1302. (B, C) Quantification of the eEPSC amplitude (B) and charge transfer (C) recorded from the strains mentioned in (A). n ≥ 11 animals. Defects in eEPSCs of unc-31 mutants can be rescued by expressing UNC-31 cDNA in acetylcholine neurons. The data are presented as box-and-whisker plots, with the median (central line), 25th–75th percentile (bounds of the box), and 5th–95th percentile (whiskers) indicated. One-way ANOVA was used for comparisons of multiple groups (F(2, 35) = 19.05, P < 0.0001 for B; F(2, 35) = 42.14, P < 0.0001 for C), followed by Tukey’s range test, * P < 0.05; ** P < 0.01; *** P < 0.001. The error bars represent the SEM. N = 3 independent replicates. All the raw data associated with this figure are available in S1 Data. (PDF) [file pbio.3003023.s002.pdf]

**A**

wild type

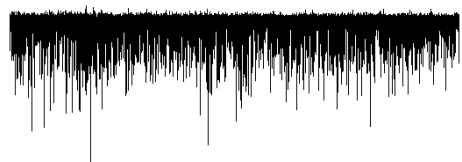*kpc-1(tm1104)*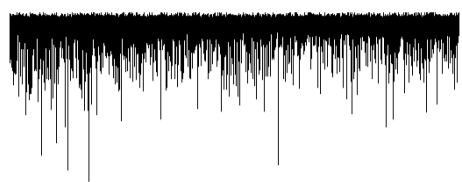*aex-5(sa23)*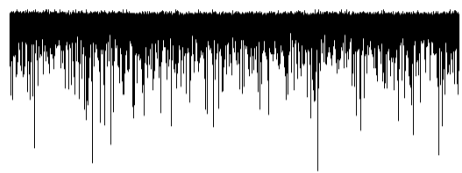*bli-4(e937)*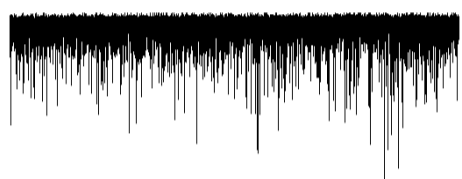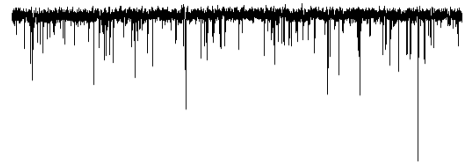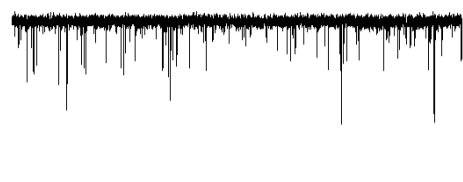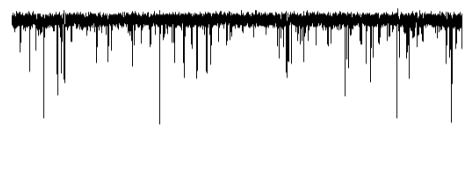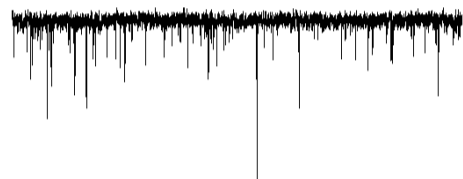50 pA  
5 s (0.5 s)**D**

wild type

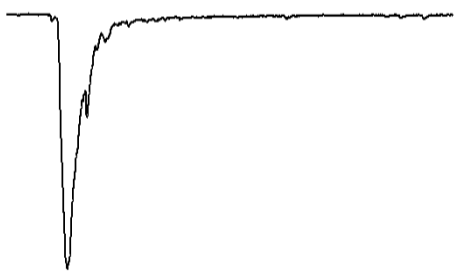*kpc-1(tm1104)*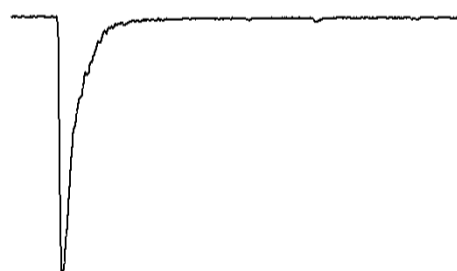*aex-5(sa23)*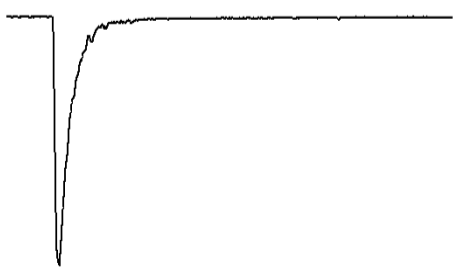*bli-4(e937)*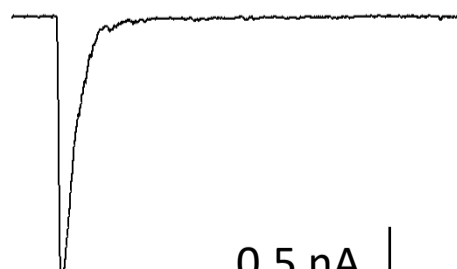0.5 nA  
30 ms**B**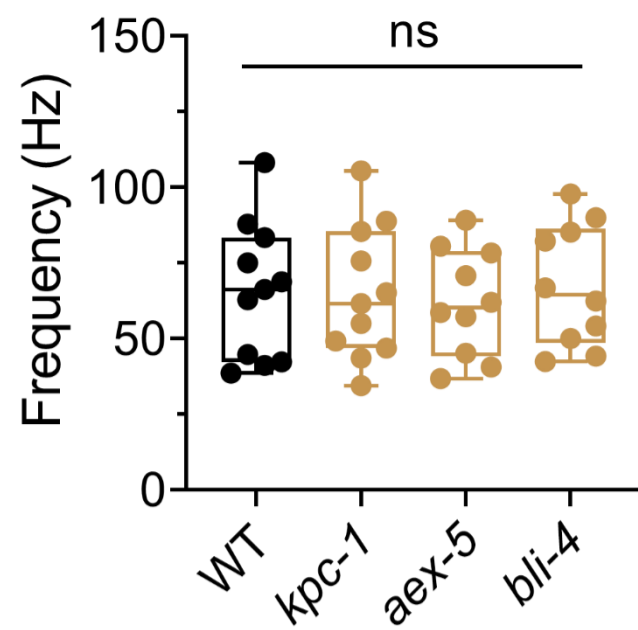**C**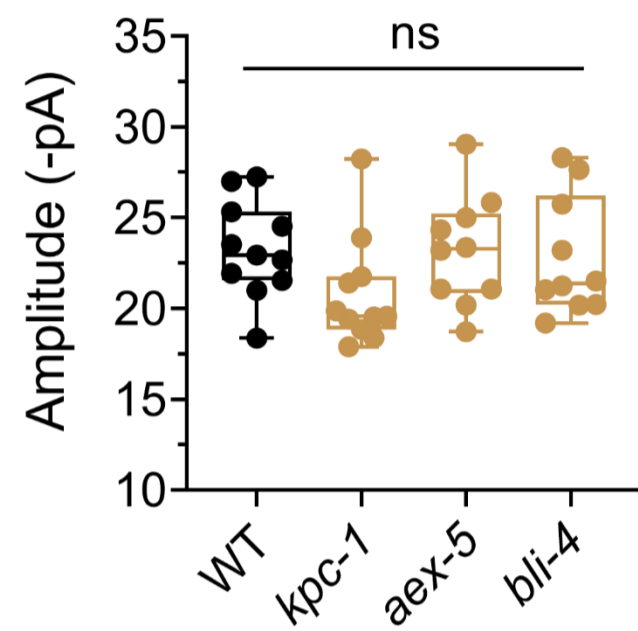**E**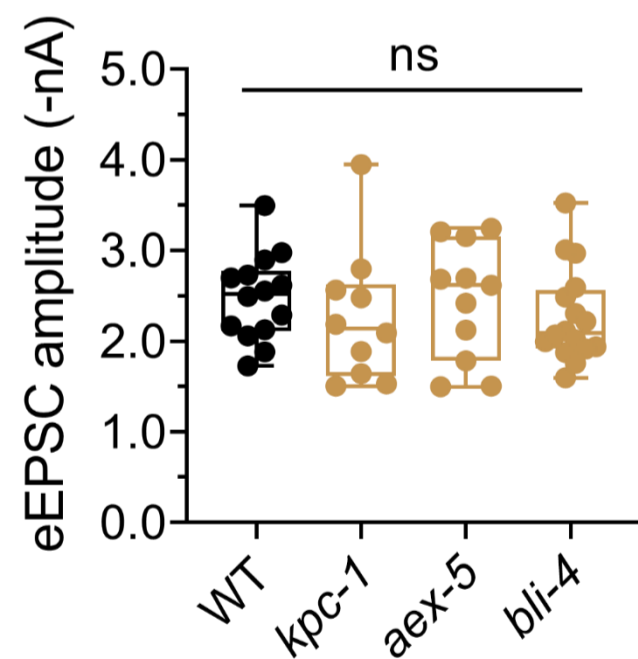**F**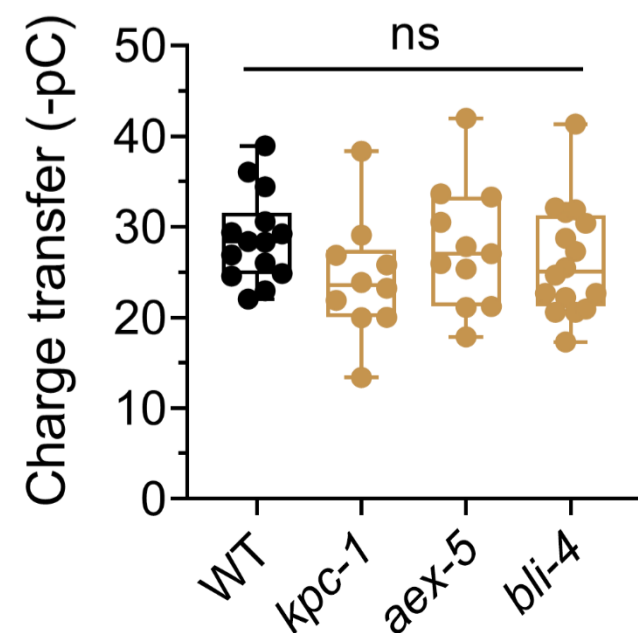

Supplement: S3 Fig — (A) Representative mPSC traces recorded from WT, kpc-1(tm1104), aex-5(sa23), and bli-4(e937) worms. The right panels show a 0.5 s scale bar for clarity. (B, C) Quantification of the mPSC frequency (B) and amplitude (C) across the indicated genotypes, as shown in panel (A). n ≥ 10 animals. One-way ANOVA was performed (F(3, 38) = 0.1245, P = 0.945 for B; F(3, 38) = 1.652, P = 0.1936 for C). (D–F) Representative traces and quantification of evoked EPSCs recorded from the mentioned genotypes. n ≥ 10 animals. One-way ANOVA was performed (F(3, 47) = 0.4829, P = 0.6958 for E; F(3, 47) = 1.201, P = 0.3197 for F). The data are presented as box-and-whisker plots, with the median (central line), 25th–75th percentile (bounds of the box), and 5th–95th percentile (whiskers) indicated. One-way ANOVA was performed; ns, not significant. The error bars represent the SEM. N = 3 independent replicates. All the raw data associated with this figure are available in S1 Data. (PDF) [file pbio.3003023.s003.pdf]

**A**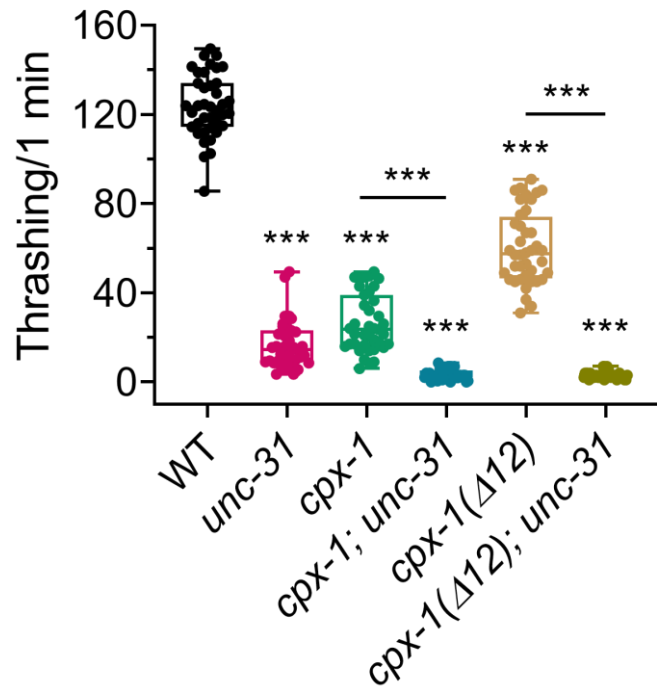**B**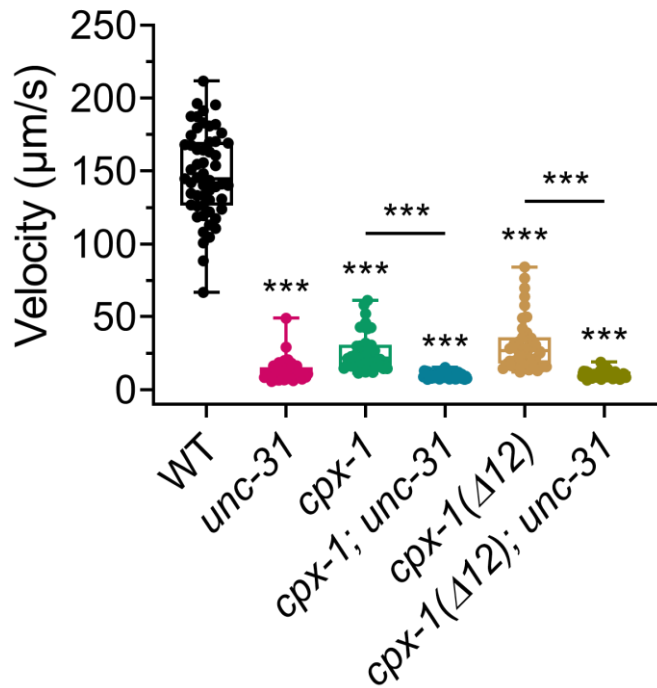

Supplement: S4 Fig — (A) Motility measured by thrashing number per minute in wild-type, unc-31(e928), cpx-1(ok1552), cpx-1(ok1552); unc-31(e928), cpx-1(Δ12), and cpx-1(Δ12); unc-31(e928) worms. n ≥ 39 animals. (B) Quantification of locomotion velocities in each genotype. n ≥ 39 animals. One-way ANOVA was performed (F(5, 230) = 673.8, P < 0.0001 for A; F(5, 263) = 566.6, P < 0.0001 for B), *** P < 0.001. The data are presented as the means ± SEMs. N = 3 independent replicates. All the raw data associated with this figure are available in S1 Data. (PDF) [file pbio.3003023.s004.pdf]

**A**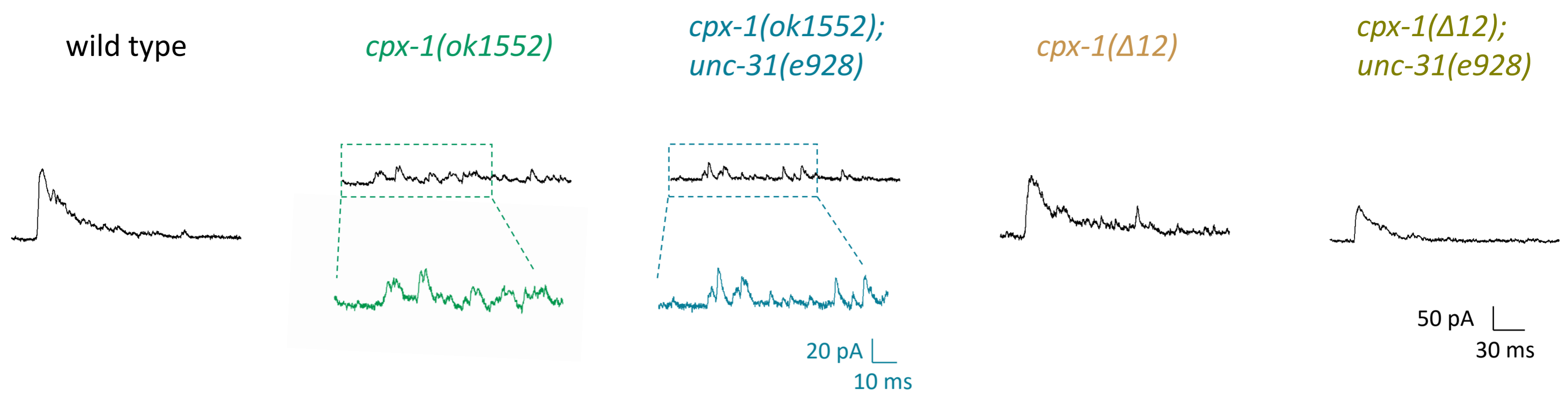**B**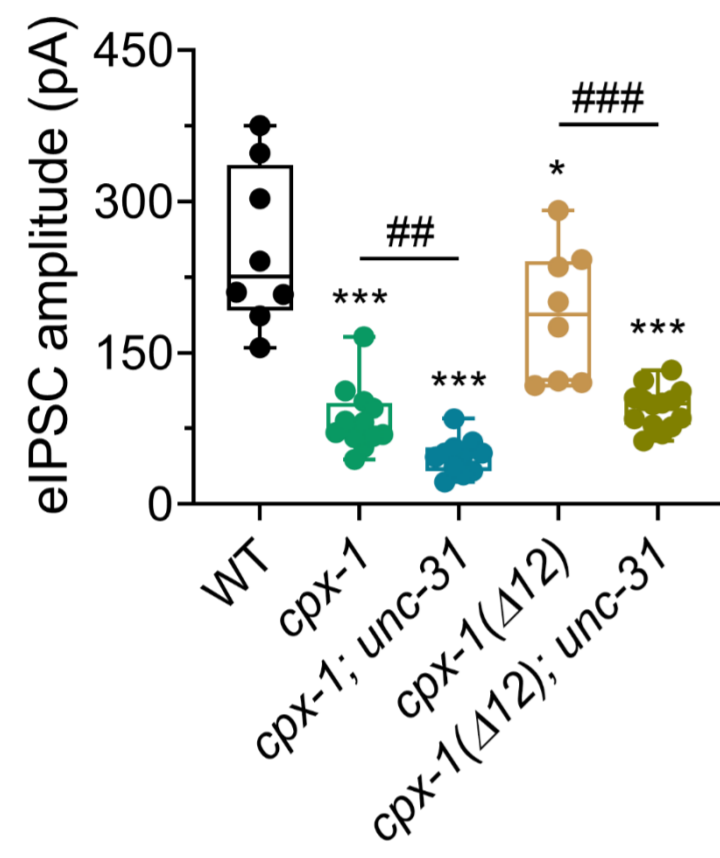**C**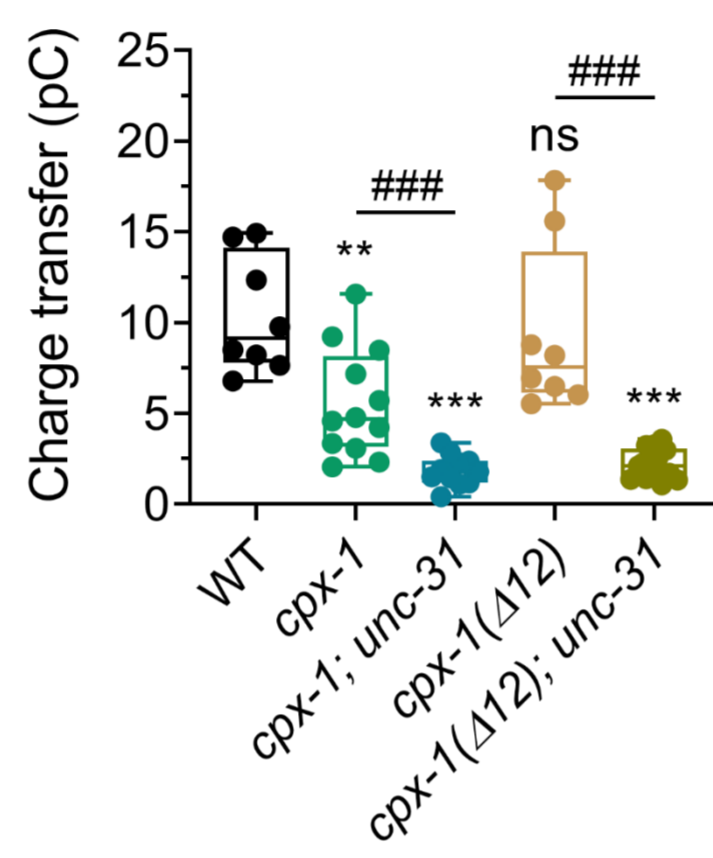**D**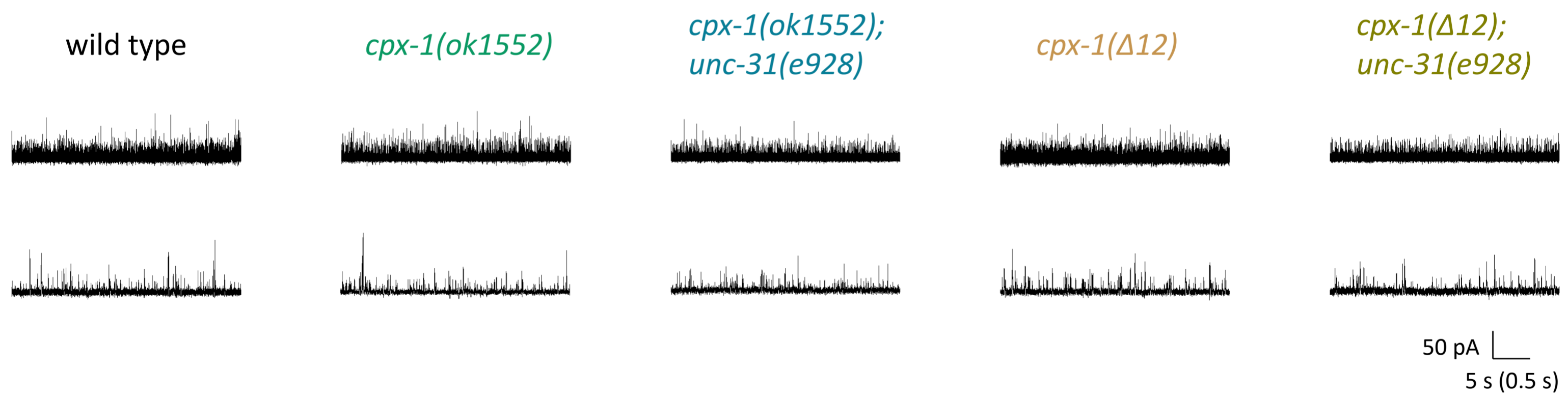**E**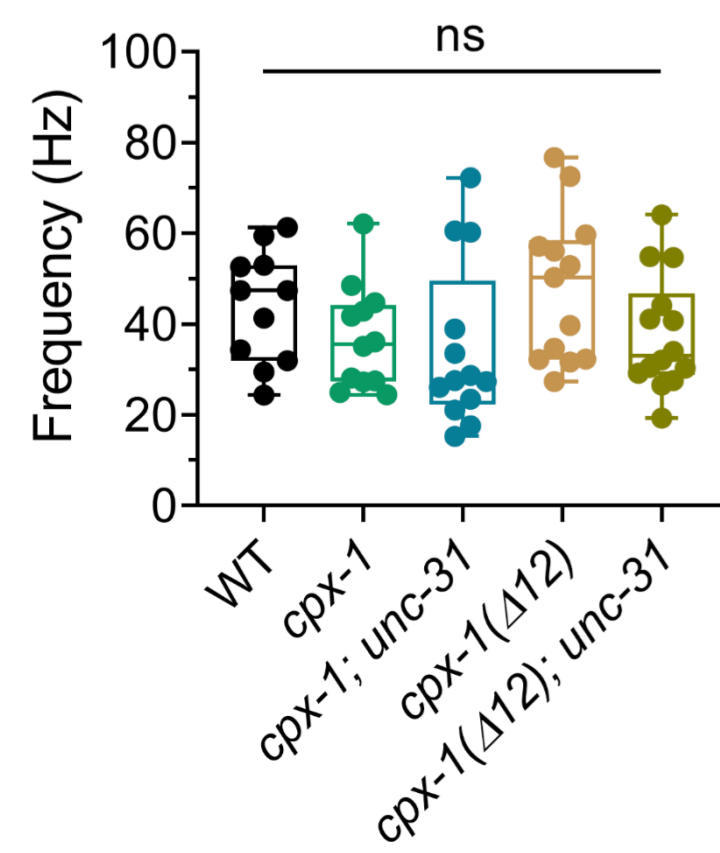**F**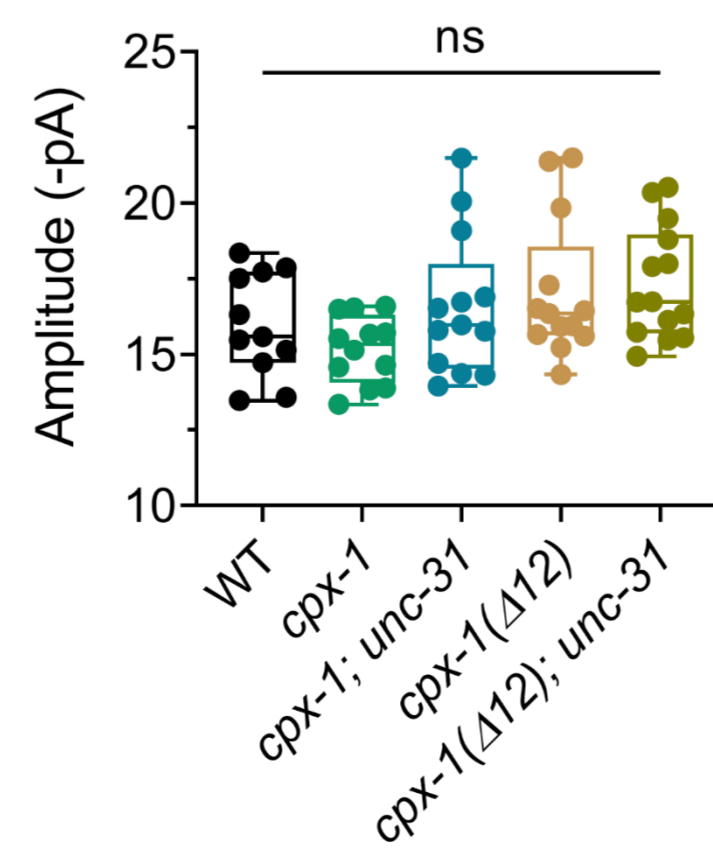

Supplement: S5 Fig — (A) Representative traces of eIPSCs recorded from the indicated strains. The lower traces of cpx-1(ok1552) (green) and cpx-1(ok1552); unc-31(e928) (blue) show a zoomed-in region from the upper traces. (B, C) Quantification of the eIPSC amplitude (B) and charge transfer (C) at each strain. n ≥ 8 animals. One-way ANOVA was performed (F(4, 48) = 33.35, P < 0.0001 for B; F(4, 48) = 21.53, P < 0.0001 for C). (D) Representative traces of mIPSCs recorded from the indicated strains at different timescales. (E, F) Quantification of the mIPSC frequency (E) and amplitude (F) at each strain. n ≥ 11 animals. One-way ANOVA was performed (F(4, 58) = 1.786, P = 0.1439 for E; F(4, 58) = 2.579, P = 0.0467 for F). The data are presented as box-and-whisker plots, with the median (central line), 25th–75th percentile (bounds of the box), and 5th–95th percentile (whiskers) indicated. Student’s t test was performed for comparisons of 2 groups, ## P < 0.01; ### P < 0.001. One-way ANOVA was used for comparisons of multiple groups, followed by Tukey’s range test, * P < 0.05; ** P < 0.01; *** P < 0.001; ns, not significant. The error bars represent the SEM. N = 3 independent replicates. All the raw data associated with this figure are available in S1 Data. (PDF) [file pbio.3003023.s005.pdf]

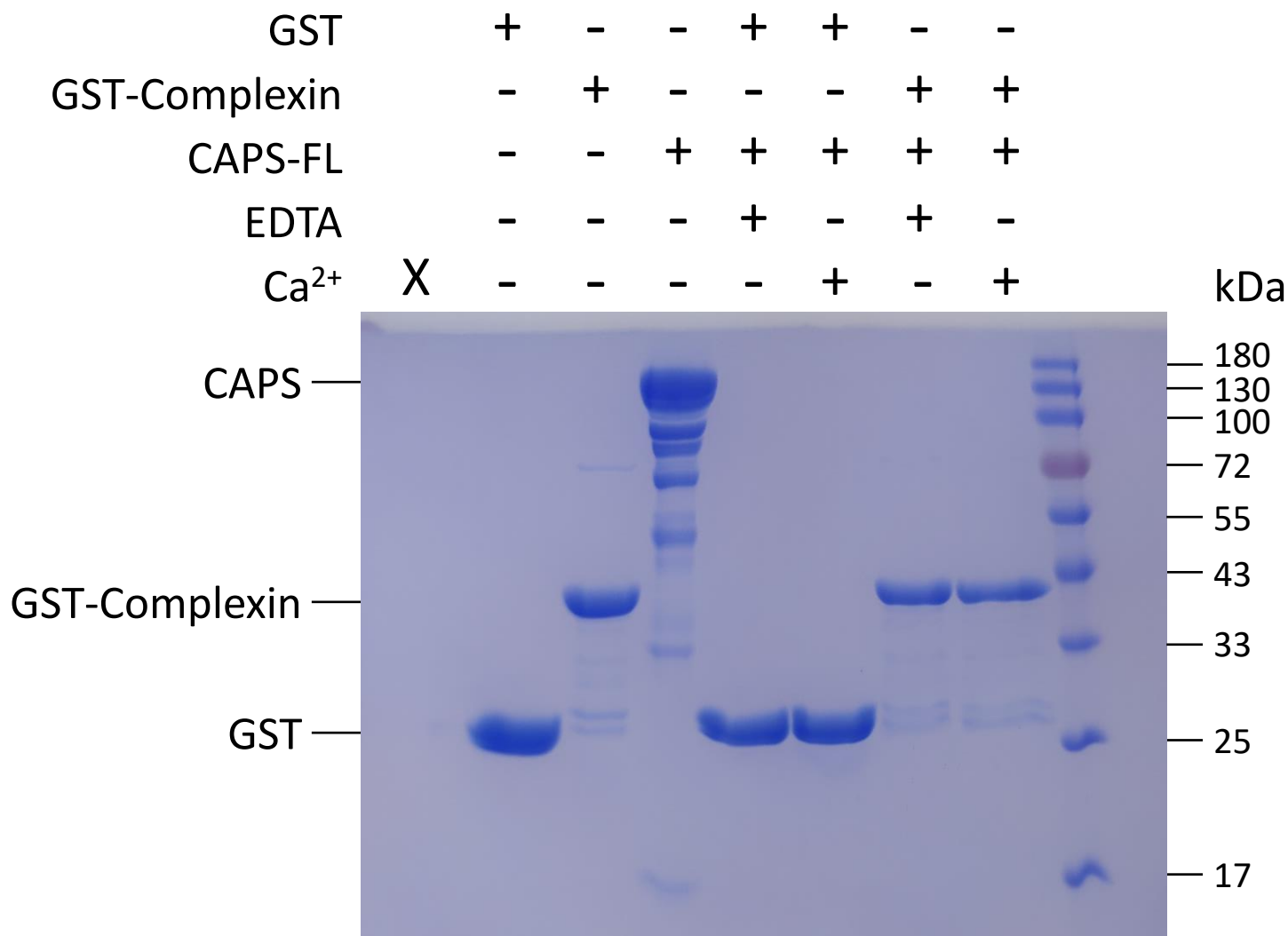

Fig. 5G

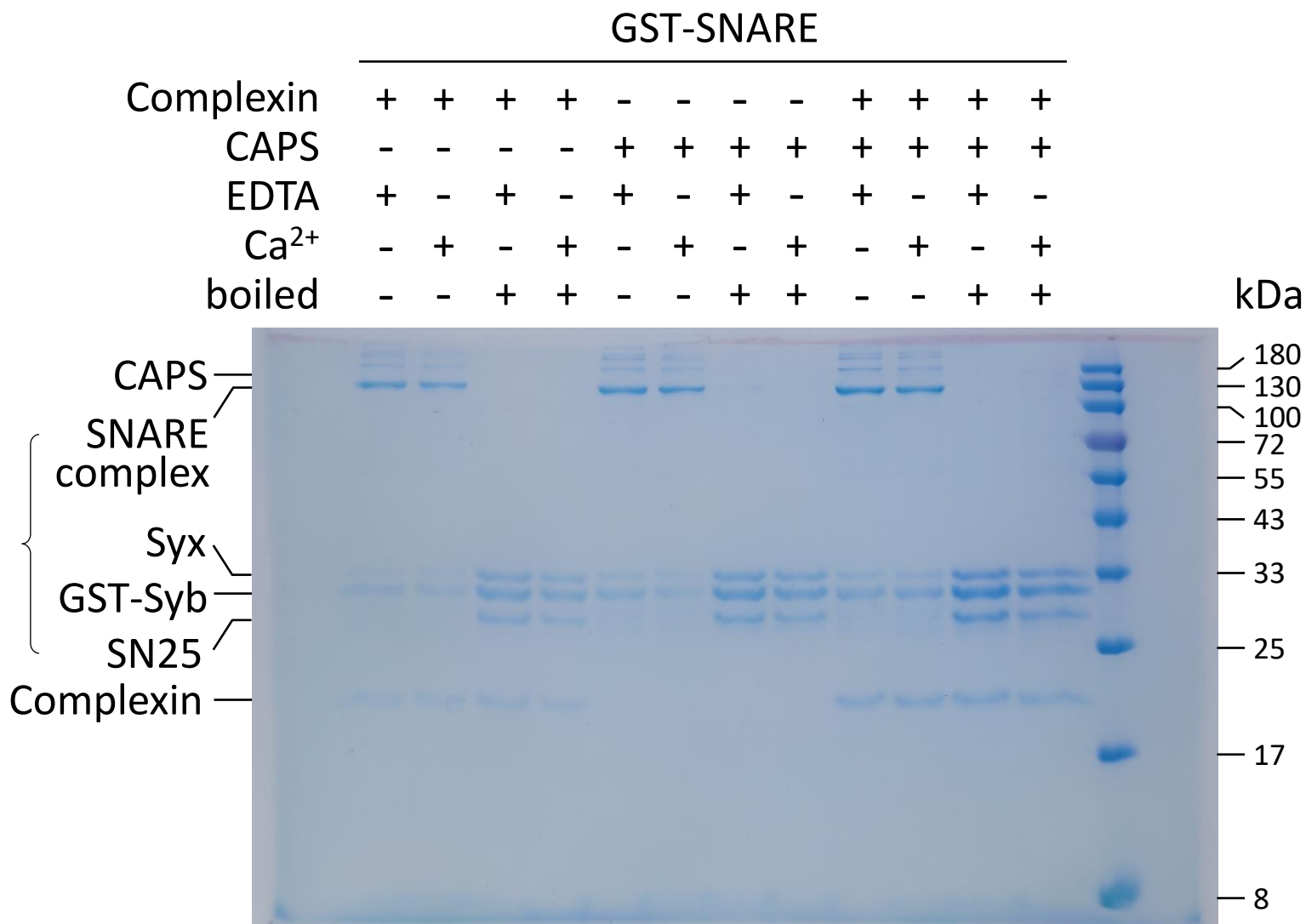

Fig. 5H

Supplement: S1 Raw Images — (PDF) [file pbio.3003023.s015.pdf]
