# Supplementary material for: The exocytosis regulator complexin controls spontaneous synaptic vesicle release in a CAPS-dependent manner at C. elegans excitatory synapses
Source: PLoS Biol. 2025 Feb 6;23(2):e3003023. doi: 10.1371/journal.pbio.3003023 (PMC11838871; doi:10.1371/journal.pbio.3003023)
Supplement: S1 Table — Genotypes of experiments in each figure. (DOCX) [file pbio.3003023.s006.docx]

**S1 Table. *C. elegans* strains used in this study.**

| **Genotype** | **Source** | **Identifier** |
| --- | --- | --- |
| N2 | *CGC* |  |
| *zxIs3* | (*57*) | ZX388 |
| *zxIs6* | (*57*) | ZX460 |
| *cpx-1(ok1552)* | *CGC* | RB1367 |
| *cpx-1(ok1552); zxIs3* | This paper | UHN69 |
| *cpx-1(ok1552); zxIs6* | This paper | UHN70 |
| *cpx-1(syb3665)* | This paper | PHX3665 |
| *cpx-1(syb3584)* | This paper | PHX3584 |
| *cpx-1(syb3584); zxIs3* | This paper | UHN71 |
| *cpx-1(syb3584); zxIs6* | This paper | UHN72 |
| *unc-31 (e928)* | *CGC* | CB928 |
| *unc-31 (e928); zxIs3* | This paper | UHN59 |
| *unc-31 (e928); zxIs6* | This paper | UHN60 |
| *egl-3 (tm1377); zxIs3* | This paper | SGA838 |
| *egl-3 (tm1377); zxIs6* | This paper | SGA713 |
| *unc-31 (e928); egl-3 (tm1377); zxIs3* | This paper | SGA839 |
| *unc-31 (e928); egl-3 (tm1377); zxIs6* | This paper | SGA714 |
| *kpc-1 (tm1104); zxIs6* | This paper | SGA882 |
| *aex-5(sa23); zxIs6* | This paper | SGA875 |
| *bli-4 (e937); zxIs6* | This paper | SGA874 |
| *cpx-1(ok1552); unc-31 (e928)* | This paper | UHN73 |
| *cpx-1(ok1552); unc-31 (e928); zxIs3* | This paper | UHN74 |
| *cpx-1(ok1552); unc-31 (e928); zxIs6* | This paper | UHN75 |
| *cpx-1(syb3584); unc-31 (e928)* | This paper | UHN76 |
| *cpx-1(syb3584); unc-31 (e928); zxIs3* | This paper | UHN77 |
| *cpx-1(syb3584); unc-31 (e928); zxIs6* | This paper | UHN78 |
| *unc-31 (e928); zxIs6; gaaEx1302[Punc-17::*  *UNC-31::wCherry; lin-44::GFP]* | This paper | SGA1050 |
