# Supplementary material for: The exocytosis regulator complexin controls spontaneous synaptic vesicle release in a CAPS-dependent manner at C. elegans excitatory synapses
Source: PLoS Biol. 2025 Feb 6;23(2):e3003023. doi: 10.1371/journal.pbio.3003023 (PMC11838871; doi:10.1371/journal.pbio.3003023)
Supplement: S2 Table — Plasmids for the generation of transgenic strains. (DOCX) [file pbio.3003023.s007.docx]

**S2 Table. List of the plasmids used in this study.**

| **Recombinant DNA** | **Source** | **Identifier** |
| --- | --- | --- |
| *pDONR221* | This paper | SG2 |
| *PDESTR4-R3II* | This paper | SG4 |
| *Plin-44::GFP* | This paper | SG5 |
| *Punc-17::unc-31::Wcherry* | This paper | SG659 |
