# Supplementary material for: The exocytosis regulator complexin controls spontaneous synaptic vesicle release in a CAPS-dependent manner at C. elegans excitatory synapses
Source: PLoS Biol. 2025 Feb 6;23(2):e3003023. doi: 10.1371/journal.pbio.3003023 (PMC11838871; doi:10.1371/journal.pbio.3003023)
Supplement: S3 Table — Primers used for PCR amplification for identification of mutants and construction of transgenic strains. (DOCX) [file pbio.3003023.s008.docx]

**S3 Table.** **List of the primers used in this study.**

| **Primer** | **Source** | **Identifier** |
| --- | --- | --- |
| 5’-CAG TCT TTC CAT CAT CAG TCG GTC-3’ | This paper | Primer 1  (*egl-3* forward) |
| 5’-GGT TAC AAG ACG AGA CGC GG-3’ | This paper | Primer 2  (*egl-3* backward) |
| 5’-CCA GAT AAT CCG GGT GAC GAG-3’ | This paper | Primer 1  (*kpc-1* forward) |
| 5’-CAG GGA AGA TGC GTA ATC CAG G-3’ | This paper | Primer 2  (*kpc-1* backward) |
| 5’-ATG ACA TTG TTC CCA CCA C-3’ | This paper | Primer 1  (*aex-5* forward) |
| 5’-CCA CTG GCC TAG AAT CCA C-3’ | This paper | Primer 2  (*aex-5* backward) |
| 5’-CAG GTA CGT TCC ACG ATT GGA C-3’ | This paper | Primer 1  (*bli-4* forward) |
| 5’-GAC GGT AGT GTT GCG AAC TGG C-3’ | This paper | Primer 2  (*bli-4* backward) |
| 5’-AAC CGC CGA ATT TAA AGT ATT G-3’ | This paper | Primer 1  (*unc-31* forward) |
| 5’-GCG CAG AGA GTT CTG AAT TTT T-3’ | This paper | Primer 2  (*unc-31* backward – wildtype specific) |
| 5’-CTG AGG GCA CGA CTC TGT TTA CTA-3’ | This paper | Primer 3  (*unc-31* backward – mutant specific) |
| 5’-CCG TGT GGA GTA CAC GAG TTT-3’ | This paper | Primer 1  (*cpx-1(ok1552)* forward) |
| 5’-CAG AAA AGA TTT TGA TGG GAA A-3’ | This paper | Primer 2  (*cpx-1(ok1552)* backward – wildtype specific) |
| 5’-GCT TTT TCA CGG ACA AAA ATT C-3’ | This paper | Primer 3  (*cpx-1(ok1552)* backward – mutant specific) |
| 5’-AAG TCC AAT CCT CTG ACC CA-3’ | This paper | Primer 1  (*cpx-1(syb3584)* forward) |
| 5’-AAC CGT AGT GAG ACA GCC TG-3’ | This paper | Primer 2  (*cpx-1(syb3584)* backward) |
| 5’-TCA CAC ACA CAC AAG ACG-3’ | This paper | Primer 1  (*cpx-1(syb3665)* forward) |
| 5’-TTT AGG AGC AAA AGA GCA CA-3’ | This paper | Primer 2  (*cpx-1(syb3665)* backward) |
| 5’-TTC GAA GGA GGT GGA AGC TTC TTC  TTC TCC-3’ | This paper | Primer 1  (*unc-17* promoter forward) |
| 5’-CTC TCT CTC TCC CCC TGG AAT ATT TTA TTT-3’ | This paper | Primer 2  (*unc-17* promoter backward) |
| 5’- ATG TTA GGA GCA AGT AGT AGT GAA  GAA GAA-3’ | This paper | Primer 1  (*unc-31* cDNA forward) |
| 5’- GCG AGA ACA TCT TTG CAC CGG CTC  CTT-3’ | This paper | Primer 2  (*unc-31* cDNA backward) |
